# (1*S*,1′*S*,2′*R*,4a’*S*,9a’*S*,9b’*R*)-1′-Acet­yloxy-2,4′-dioxo-2′,4′,4a’,7′,8′,9′,9a’,9b’-octa­hydro-1′*H*,2*H*-spiro­[ace­naphthyl­ene-1,5′-pyrano[4,3-*a*]pyrrolizin]-2′-ylmethyl acetate

**DOI:** 10.1107/S1600536813026111

**Published:** 2013-10-02

**Authors:** S. Santhiya, J. Naga Siva Rao, R. Raghunathan, N. Latha, S. Lakshmi

**Affiliations:** a Research Department of Physics, SDNB Vaishnav College for Women, Chennai 600 004, India; b Department of Organic Chemistry, University of Madras, Guindy, Chennai 600 025, India

## Abstract

In the title compound C_26_H_25_NO_7_, the mean plane through the lactone-substituted ring of the pyrrolizidine moiety forms dihedral angles of 78.46 (6) and 58.28 (8)° with the ace­naphthyl­ene moiety and the sugar based-lactone ring, respectively. The sum of the angles at the the N atom of the pyrrolizidine ring (335.0°) is in accordance with *sp* ^3^ hybridization. Some atoms of the acetate group are disordered and were refined using a split model [occupancy ratio 0.673 (10):0.327 (10)].

## Related literature

For the importance of pyrrolidine and pyrrolizidine compounds and background to this work, see: Boido *et al.* (1994 ▶); Cravotto *et al.* (2001 ▶); Gershbein (1975 ▶); Govind *et al.* (2003 ▶); Jellimann *et al.* (2000 ▶); Nishimura *et al.* (1985 ▶); Selvanayagam *et al.* (2004 ▶); Usha *et al.* (2005 ▶).
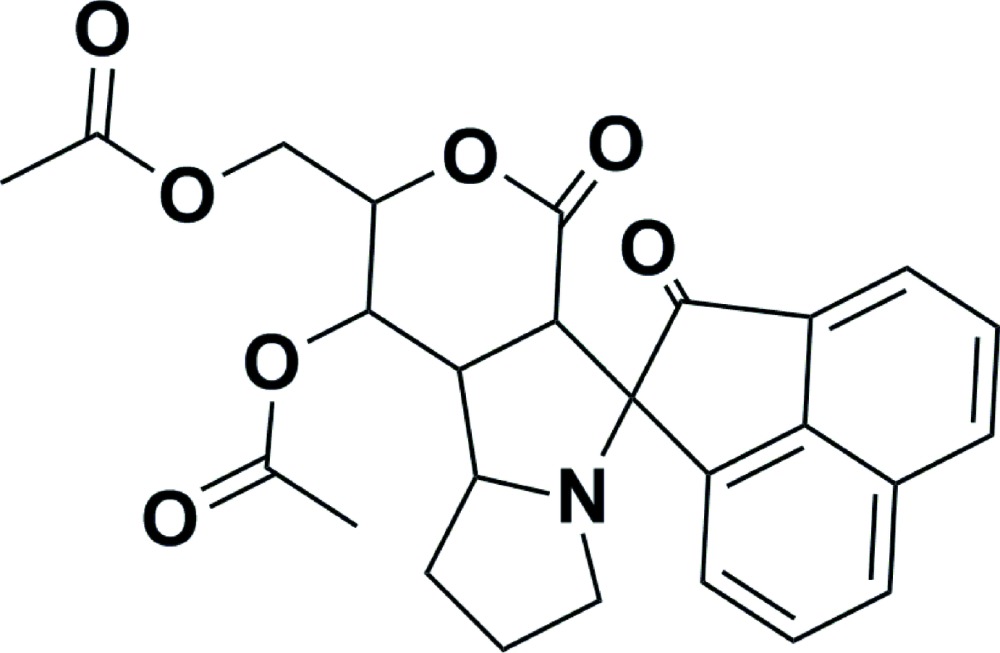


## Experimental

### 

#### Crystal data

C_26_H_25_NO_7_
*M* *_r_* = 463.47
Tetragonal, 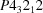

*a* = 13.6792 (3) Å
*c* = 24.9625 (13) Å
*V* = 4671.0 (3) Å^3^
*Z* = 8
Mo *K*α radiation
μ = 0.10 mm^−1^
*T* = 295 K
0.30 × 0.30 × 0.25 mm

#### Data collection

Bruker Kappa APEXII CCD diffractometer
Absorption correction: multi-scan (*SADABS*; Bruker, 2004 ▶) *T*_min_ = 0.902, *T*_max_ = 0.976
21173 measured reflections
4010 independent reflections
2923 reflections with *I* > 2σ(*I*)
*R*_int_ = 0.034

#### Refinement

*R*[*F* ^2^ > 2σ(*F* ^2^)] = 0.042
*wR*(*F* ^2^) = 0.104
*S* = 1.06
4010 reflections
367 parameters
138 restraints
H-atom parameters constrained
Δρ_max_ = 0.24 e Å^−3^
Δρ_min_ = −0.12 e Å^−3^

### 

Data collection: *APEX2* (Bruker, 2004 ▶); cell refinement: *APEX2* and *SAINT* (Bruker, 2004 ▶); data reduction: *SAINT* and *XPREP* (Bruker, 2004 ▶); program(s) used to solve structure: *SIR92* (Altomare *et al.*, 1993 ▶); program(s) used to refine structure: *SHELXL97* (Sheldrick, 2008 ▶); molecular graphics: *ORTEP-3 for Windows* (Farrugia, 2012 ▶); software used to prepare material for publication: *PLATON* (Spek, 2009 ▶).

## Supplementary Material

Crystal structure: contains datablock(s) I, New_Global_Publ_Block. DOI: 10.1107/S1600536813026111/nc2316sup1.cif

Structure factors: contains datablock(s) I. DOI: 10.1107/S1600536813026111/nc2316Isup2.hkl

Additional supplementary materials:  crystallographic information; 3D view; checkCIF report

## Figures and Tables

**Fig. 1.**
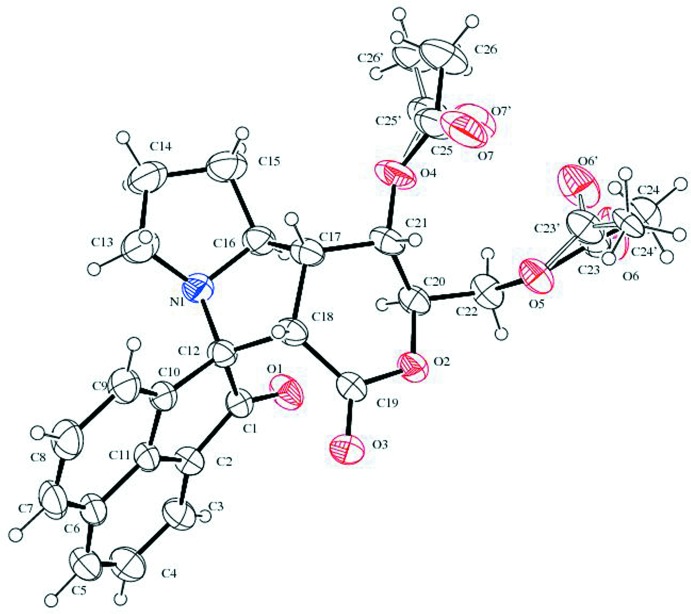
The molecular structure of the title compound with the numbering scheme. Displacement ellipsoids are drawn at the 50% probability level. H atoms are presented as small spheres of arbitrary radius and disordering is shown with full and open bonds.

## supplementary crystallographic information

### 1. Comment

Synthesis of polycyclic compounds incorporating pyrrolidine and pyrrolizidine rings has been the center of attraction for the past several decades since it constitutes significant class of substances with highly pronounced biological activities (Nishimura *et al.*,1985). Pyrrolizidine alkaloids represent a group of compounds present in a variety of plants throughout the world (Usha *et al.*, 2005). Acenaphthylene derivatives are found to have high κ-opioid receptor affinity and selectivity (Selvanayagam *et al.*, 2004). These derivatives are used as new conformationally restricted ligands for melatonin receptors (Jellimann *et al.*, 2000), liver regeneration (Gershbein *et al.*, 1975) and antitumoral agents (Boido *et al.*, 1994). Spiro compounds are often encountered in pharmacologically relevant alkaloids (Cravotto *et al.*, 2001). In view of the important biological activities, the structure determination of the title compound was undertaken (Fig.1).

The lactone substituted ring of the pyrrolizidine moiety [C12/N1/C16—C18] forms dihedral angles of 78.46 (6)°, 58.28 (8)° with the acenaphthylene moiety [C12/C1—C11] and sugar based-lactone ring [C18/C17/C21/C20/O2/C19] respectively. The sum of the angles at nitrogen atom of the pyrrolizidine ring [335.01 (0.6)°] is in accordance with *sp*^3^ hybridization (Govind *et al.*,2003).

### 2. Experimental

To a solution of acenaphthoquinone (1 equiv) and proline (1.4 equiv) in dry toluene, α,β- unsaturated sugar lactone was added under nitrogen atmosphere. The solution was refluxed for 10 h until the reaction was complete which was monitored by TLC. The solvent was evaporated under reduced pressure and the residue was extracted with dichloromethane and water. The organic layer was dried with anhydrous sodium sulfate and concentrated *in vacuo*. The crude product was purified by column chromatography using hexane/EtOAc (8:2) as an eluent. Crystals were obtained by slow evaporation of the solvent.

### 3. Refinement

Both acetate groups are disordered over two positions with side occupancies of 0.673 (10) and 0.327 (10) respectively. The disorder was refined using restraints for the bond distances of 1.50 (1) A° for C—C, 1.32 (1) A° and 1.20 (1) A° for C—O and C=O respectively. Restraints were also used to refine the atomic displacement parameters. The hydrogen atoms were placed in calculated positions and were refined with *U*_iso_(H) = 1.2*U*_eq_(C) for non methyl group and *U*_iso_(H) = 1.5*U*_eq_(C) for methyl group using a riding model.The absolute structure was not determined since no strong anomalous scattering atoms are present.

### Crystal data

**Table:**

C_26_H_25_NO_7_	*D*_x_ = 1.318 Mg m^−^^3^
*M**_r_* = 463.47	Melting point: 481.15 K
Tetragonal, *P*4_3_2_1_2	Mo *K*α radiation, λ = 0.71073 Å
Hall symbol: P4nw2abw	Cell parameters from 5405 reflections
*a* = 13.6792 (3) Å	θ = 2.2–22.2°
*c* = 24.9625 (13) Å	µ = 0.10 mm^−^^1^
*V* = 4671.0 (3) Å^3^	*T* = 295 K
*Z* = 8	Block, yellow
*F*(000) = 1952	0.30 × 0.30 × 0.25 mm

### Data collection

**Table:**

Bruker Kappa APEXII CCD diffractometer	4010 independent reflections
Radiation source: fine-focus sealed tube	2923 reflections with *I* > 2σ(*I*)
Graphite monochromator	*R*_int_ = 0.034
ω and φ scan	θ_max_ = 25.1°, θ_min_ = 2.1°
Absorption correction: multi-scan (*SADABS*; Bruker, 2004)	*h* = −16→16
*T*_min_ = 0.902, *T*_max_ = 0.976	*k* = −11→16
21173 measured reflections	*l* = −27→28

### Refinement

**Table:**

Refinement on *F*^2^	Secondary atom site location: difference Fourier map
Least-squares matrix: full	Hydrogen site location: inferred from neighbouring sites
*R*[*F*^2^ > 2σ(*F*^2^)] = 0.042	H-atom parameters constrained
*wR*(*F*^2^) = 0.104	*w* = 1/[σ^2^(*F*_o_^2^) + (0.0494*P*)^2^ + 0.438*P*] where *P* = (*F*_o_^2^ + 2*F*_c_^2^)/3
*S* = 1.06	(Δ/σ)_max_ < 0.001
4010 reflections	Δρ_max_ = 0.24 e Å^−^^3^
367 parameters	Δρ_min_ = −0.12 e Å^−^^3^
138 restraints	Extinction correction: *SHELXL97* (Sheldrick, 2008), Fc^*^=kFc[1+0.001xFc^2^λ^3^/sin(2θ)]^-1/4^
Primary atom site location: structure-invariant direct methods	Extinction coefficient: 0.0038 (5)

### Special details

**Table:**

Geometry. All e.s.d.'s (except the e.s.d. in the dihedral angle between two l.s. planes) are estimated using the full covariance matrix. The cell e.s.d.'s are taken into account individually in the estimation of e.s.d.'s in distances, angles and torsion angles; correlations between e.s.d.'s in cell parameters are only used when they are defined by crystal symmetry. An approximate (isotropic) treatment of cell e.s.d.'s is used for estimating e.s.d.'s involving l.s. planes.
Refinement. Refinement of *F*^2^ against ALL reflections. The weighted *R*-factor *wR* and goodness of fit *S* are based on *F*^2^, conventional *R*-factors *R* are based on *F*, with *F* set to zero for negative *F*^2^. The threshold expression of *F*^2^ > σ(*F*^2^) is used only for calculating *R*-factors(gt) *etc*. and is not relevant to the choice of reflections for refinement. *R*-factors based on *F*^2^ are statistically about twice as large as those based on *F*, and *R*- factors based on ALL data will be even larger.

### Fractional atomic coordinates and isotropic or equivalent isotropic displacement parameters (Å^2^)

**Table:**

	*x*	*y*	*z*	*U*_iso_*/*U*_eq_	Occ. (<1)
C1	0.49450 (18)	0.37963 (17)	0.34572 (10)	0.0529 (6)	
C2	0.52973 (19)	0.38194 (18)	0.40078 (10)	0.0567 (7)	
C3	0.5995 (2)	0.4377 (2)	0.42606 (12)	0.0727 (8)	
H3	0.6377	0.4823	0.4073	0.087*	
C4	0.6103 (2)	0.4243 (3)	0.48143 (14)	0.0894 (10)	
H4	0.6574	0.4609	0.4994	0.107*	
C5	0.5552 (3)	0.3604 (3)	0.51006 (13)	0.0843 (9)	
H5	0.5649	0.3551	0.5468	0.101*	
C6	0.4840 (2)	0.3023 (2)	0.48530 (10)	0.0650 (7)	
C7	0.4197 (2)	0.2338 (2)	0.50876 (12)	0.0785 (9)	
H7	0.4215	0.2233	0.5456	0.094*	
C8	0.3560 (2)	0.1837 (2)	0.47842 (11)	0.0763 (8)	
H8	0.3157	0.1380	0.4949	0.092*	
C9	0.3474 (2)	0.19735 (19)	0.42248 (11)	0.0660 (7)	
H9	0.3017	0.1621	0.4028	0.079*	
C10	0.40725 (18)	0.26315 (17)	0.39801 (9)	0.0522 (6)	
C11	0.47403 (18)	0.31481 (17)	0.42968 (9)	0.0538 (6)	
C12	0.41909 (17)	0.29582 (17)	0.34043 (9)	0.0508 (6)	
C13	0.4556 (3)	0.1226 (2)	0.31434 (13)	0.0898 (10)	
H13A	0.3875	0.1058	0.3201	0.108*	
H13B	0.4939	0.1007	0.3448	0.108*	
C14	0.4941 (3)	0.0793 (2)	0.26195 (13)	0.0945 (11)	
H14A	0.5650	0.0791	0.2612	0.113*	
H14B	0.4704	0.0131	0.2567	0.113*	
C15	0.4528 (3)	0.1477 (2)	0.22047 (12)	0.0893 (10)	
H15A	0.3920	0.1224	0.2061	0.107*	
H15B	0.4987	0.1568	0.1913	0.107*	
C16	0.4355 (2)	0.24436 (19)	0.25035 (10)	0.0639 (7)	
H16	0.4763	0.2958	0.2346	0.077*	
C17	0.33003 (19)	0.28012 (19)	0.25462 (9)	0.0587 (7)	
H17	0.2867	0.2230	0.2540	0.070*	
C18	0.32353 (17)	0.32766 (17)	0.31052 (9)	0.0505 (6)	
H18	0.2686	0.2968	0.3292	0.061*	
C19	0.30811 (17)	0.43601 (18)	0.31357 (10)	0.0532 (6)	
C20	0.34631 (19)	0.44821 (18)	0.21942 (9)	0.0567 (6)	
H20	0.4167	0.4371	0.2235	0.068*	
C21	0.2963 (2)	0.3508 (2)	0.21183 (9)	0.0607 (7)	
H21	0.2252	0.3589	0.2135	0.073*	
C22	0.33054 (19)	0.5216 (2)	0.17621 (10)	0.0660 (7)	
H22A	0.3560	0.5847	0.1873	0.079*	
H22B	0.3646	0.5016	0.1439	0.079*	
N1	0.46742 (16)	0.22513 (15)	0.30539 (8)	0.0636 (6)	
O1	0.51791 (14)	0.43233 (14)	0.30886 (7)	0.0734 (6)	
O2	0.30863 (12)	0.49008 (12)	0.26880 (6)	0.0599 (4)	
O3	0.29131 (15)	0.47673 (14)	0.35530 (7)	0.0734 (5)	
O5	0.22688 (12)	0.52899 (14)	0.16582 (7)	0.0674 (5)	
O4	0.32451 (17)	0.31189 (15)	0.16054 (7)	0.0873 (7)	
O6	0.2533 (3)	0.5940 (6)	0.08779 (18)	0.108 (2)	0.673 (10)
C23	0.1979 (6)	0.5579 (7)	0.1189 (2)	0.066 (2)	0.673 (10)
C24	0.0924 (7)	0.5520 (13)	0.1066 (6)	0.082 (3)	0.673 (10)
H24A	0.0823	0.5635	0.0691	0.123*	0.673 (10)
H24B	0.0579	0.6005	0.1270	0.123*	0.673 (10)
H24C	0.0685	0.4882	0.1158	0.123*	0.673 (10)
O6'	0.2525 (6)	0.5122 (12)	0.0794 (3)	0.101 (4)	0.327 (10)
C23'	0.1997 (12)	0.5354 (18)	0.1156 (4)	0.073 (4)	0.327 (10)
C24'	0.0906 (13)	0.540 (3)	0.1196 (11)	0.072 (5)	0.327 (10)
H24D	0.0625	0.4829	0.1039	0.109*	0.327 (10)
H24E	0.0674	0.5973	0.1009	0.109*	0.327 (10)
H24F	0.0718	0.5444	0.1566	0.109*	0.327 (10)
O7	0.1627 (7)	0.3021 (9)	0.1393 (4)	0.114 (3)	0.673 (10)
C25	0.2479 (7)	0.2915 (16)	0.1279 (5)	0.105 (3)	0.673 (10)
C26	0.2787 (9)	0.2525 (11)	0.0740 (5)	0.143 (4)	0.673 (10)
H26A	0.3158	0.1936	0.0787	0.215*	0.673 (10)
H26B	0.3182	0.3003	0.0560	0.215*	0.673 (10)
H26C	0.2217	0.2388	0.0528	0.215*	0.673 (10)
O7'	0.1851 (18)	0.3144 (19)	0.1230 (9)	0.122 (5)	0.327 (10)
C25'	0.2665 (17)	0.282 (3)	0.1209 (12)	0.112 (4)	0.327 (10)
C26'	0.3264 (19)	0.2324 (18)	0.0783 (9)	0.119 (6)	0.327 (10)
H26D	0.2952	0.1724	0.0680	0.178*	0.327 (10)
H26E	0.3905	0.2186	0.0921	0.178*	0.327 (10)
H26F	0.3318	0.2745	0.0477	0.178*	0.327 (10)

### Atomic displacement parameters (Å^2^)

**Table:**

	*U*^11^	*U*^22^	*U*^33^	*U*^12^	*U*^13^	*U*^23^
C1	0.0599 (16)	0.0540 (15)	0.0449 (16)	0.0000 (11)	0.0005 (12)	0.0049 (12)
C2	0.0620 (16)	0.0603 (16)	0.0476 (16)	0.0012 (12)	−0.0052 (13)	−0.0022 (12)
C3	0.0722 (19)	0.081 (2)	0.065 (2)	−0.0042 (15)	−0.0190 (15)	−0.0035 (15)
C4	0.093 (2)	0.097 (3)	0.078 (3)	−0.0003 (19)	−0.0338 (19)	−0.0095 (19)
C5	0.104 (3)	0.095 (2)	0.0541 (19)	0.026 (2)	−0.0200 (18)	−0.0050 (17)
C6	0.083 (2)	0.0668 (17)	0.0449 (17)	0.0219 (14)	−0.0037 (15)	0.0061 (14)
C7	0.105 (2)	0.084 (2)	0.0463 (18)	0.0269 (18)	0.0066 (18)	0.0209 (16)
C8	0.100 (2)	0.0702 (19)	0.059 (2)	0.0063 (16)	0.0176 (17)	0.0228 (16)
C9	0.0806 (19)	0.0600 (17)	0.0573 (18)	−0.0029 (13)	0.0101 (14)	0.0101 (13)
C10	0.0684 (16)	0.0467 (14)	0.0416 (15)	0.0005 (11)	0.0042 (12)	0.0068 (11)
C11	0.0700 (16)	0.0531 (14)	0.0382 (15)	0.0124 (12)	0.0000 (12)	0.0057 (11)
C12	0.0618 (15)	0.0496 (14)	0.0410 (15)	−0.0057 (11)	0.0025 (11)	0.0001 (11)
C13	0.120 (3)	0.066 (2)	0.084 (2)	0.0018 (17)	0.0054 (19)	−0.0087 (17)
C14	0.128 (3)	0.0651 (19)	0.091 (2)	0.0087 (19)	0.010 (2)	−0.0184 (18)
C15	0.113 (3)	0.082 (2)	0.072 (2)	0.0072 (19)	0.0118 (18)	−0.0235 (17)
C16	0.083 (2)	0.0637 (17)	0.0446 (17)	−0.0005 (13)	0.0097 (13)	−0.0034 (12)
C17	0.0706 (17)	0.0619 (16)	0.0438 (16)	−0.0159 (12)	0.0010 (12)	−0.0035 (12)
C18	0.0568 (15)	0.0555 (15)	0.0392 (15)	−0.0135 (10)	0.0036 (11)	0.0000 (11)
C19	0.0559 (15)	0.0629 (17)	0.0409 (16)	−0.0026 (11)	−0.0021 (12)	0.0011 (13)
C20	0.0592 (15)	0.0716 (17)	0.0392 (15)	−0.0074 (12)	0.0031 (11)	0.0029 (12)
C21	0.0710 (17)	0.0756 (18)	0.0357 (15)	−0.0145 (14)	−0.0006 (12)	−0.0027 (13)
C22	0.0589 (17)	0.0831 (19)	0.0559 (17)	−0.0138 (13)	−0.0011 (13)	0.0119 (14)
N1	0.0864 (16)	0.0562 (14)	0.0481 (14)	0.0016 (11)	0.0048 (11)	−0.0009 (10)
O1	0.0849 (13)	0.0802 (13)	0.0552 (12)	−0.0292 (10)	−0.0069 (10)	0.0176 (10)
O2	0.0759 (12)	0.0608 (11)	0.0430 (10)	−0.0020 (8)	−0.0031 (8)	0.0035 (8)
O3	0.1011 (14)	0.0731 (12)	0.0459 (11)	0.0151 (10)	0.0021 (9)	−0.0077 (10)
O5	0.0635 (12)	0.0933 (13)	0.0453 (11)	−0.0059 (9)	−0.0001 (9)	0.0081 (9)
O4	0.1211 (18)	0.1029 (16)	0.0377 (12)	−0.0152 (13)	−0.0072 (11)	−0.0139 (11)
O6	0.092 (3)	0.157 (5)	0.075 (3)	0.024 (3)	0.018 (2)	0.060 (3)
C23	0.075 (3)	0.080 (5)	0.042 (3)	0.012 (3)	0.002 (3)	0.001 (2)
C24	0.082 (4)	0.087 (5)	0.076 (7)	0.001 (3)	−0.015 (4)	0.002 (6)
O6'	0.096 (5)	0.158 (9)	0.050 (4)	0.004 (5)	0.015 (4)	0.012 (5)
C23'	0.069 (6)	0.101 (8)	0.049 (6)	−0.010 (5)	0.000 (5)	0.001 (5)
C24'	0.083 (7)	0.089 (10)	0.046 (8)	0.010 (7)	−0.036 (6)	−0.015 (8)
O7	0.144 (5)	0.126 (4)	0.072 (5)	−0.050 (3)	−0.041 (4)	0.001 (4)
C25	0.165 (7)	0.095 (6)	0.054 (5)	−0.022 (6)	−0.024 (6)	−0.010 (4)
C26	0.214 (10)	0.159 (8)	0.056 (5)	0.001 (8)	−0.032 (6)	−0.027 (5)
O7'	0.186 (12)	0.110 (8)	0.071 (10)	−0.011 (9)	−0.041 (8)	−0.007 (7)
C25'	0.179 (8)	0.098 (7)	0.058 (7)	−0.028 (7)	−0.022 (7)	0.003 (6)
C26'	0.210 (16)	0.106 (9)	0.040 (7)	0.004 (11)	−0.027 (10)	−0.021 (6)

### Geometric parameters (Å, º)

**Table:**

C1—O1	1.212 (3)	C17—H17	0.9800
C1—C2	1.457 (3)	C18—C19	1.499 (3)
C1—C12	1.548 (3)	C18—H18	0.9800
C2—C3	1.375 (4)	C19—O3	1.203 (3)
C2—C11	1.394 (3)	C19—O2	1.340 (3)
C3—C4	1.402 (4)	C20—O2	1.454 (3)
C3—H3	0.9300	C20—C22	1.490 (3)
C4—C5	1.357 (5)	C20—C21	1.509 (4)
C4—H4	0.9300	C20—H20	0.9800
C5—C6	1.401 (4)	C21—O4	1.439 (3)
C5—H5	0.9300	C21—H21	0.9800
C6—C11	1.405 (3)	C22—O5	1.445 (3)
C6—C7	1.412 (4)	C22—H22A	0.9700
C7—C8	1.343 (4)	C22—H22B	0.9700
C7—H7	0.9300	O5—C23	1.299 (6)
C8—C9	1.414 (4)	O5—C23'	1.310 (9)
C8—H8	0.9300	O4—C25'	1.333 (10)
C9—C10	1.362 (3)	O4—C25	1.356 (7)
C9—H9	0.9300	O6—C23	1.192 (7)
C10—C11	1.400 (3)	C23—C24	1.477 (7)
C10—C12	1.514 (3)	C24—H24A	0.9600
C12—N1	1.462 (3)	C24—H24B	0.9600
C12—C18	1.567 (3)	C24—H24C	0.9600
C13—N1	1.429 (4)	O6'—C23'	1.199 (9)
C13—C14	1.529 (4)	C23'—C24'	1.498 (10)
C13—H13A	0.9700	C24'—H24D	0.9600
C13—H13B	0.9700	C24'—H24E	0.9600
C14—C15	1.505 (4)	C24'—H24F	0.9600
C14—H14A	0.9700	O7—C25	1.210 (8)
C14—H14B	0.9700	C25—C26	1.508 (8)
C15—C16	1.537 (4)	C26—H26A	0.9600
C15—H15A	0.9700	C26—H26B	0.9600
C15—H15B	0.9700	C26—H26C	0.9600
C16—N1	1.465 (3)	O7'—C25'	1.199 (10)
C16—C17	1.527 (4)	C25'—C26'	1.504 (10)
C16—H16	0.9800	C26'—H26D	0.9600
C17—C21	1.513 (3)	C26'—H26E	0.9600
C17—C18	1.542 (3)	C26'—H26F	0.9600
			
O1—C1—C2	128.0 (2)	C16—C17—C18	104.6 (2)
O1—C1—C12	123.5 (2)	C21—C17—H17	108.3
C2—C1—C12	108.5 (2)	C16—C17—H17	108.3
C3—C2—C11	120.5 (2)	C18—C17—H17	108.3
C3—C2—C1	132.4 (3)	C19—C18—C17	118.1 (2)
C11—C2—C1	107.0 (2)	C19—C18—C12	111.59 (18)
C2—C3—C4	116.9 (3)	C17—C18—C12	105.41 (19)
C2—C3—H3	121.5	C19—C18—H18	107.1
C4—C3—H3	121.5	C17—C18—H18	107.1
C5—C4—C3	123.0 (3)	C12—C18—H18	107.1
C5—C4—H4	118.5	O3—C19—O2	117.9 (2)
C3—C4—H4	118.5	O3—C19—C18	121.9 (2)
C4—C5—C6	121.3 (3)	O2—C19—C18	120.2 (2)
C4—C5—H5	119.4	O2—C20—C22	107.3 (2)
C6—C5—H5	119.4	O2—C20—C21	107.08 (18)
C5—C6—C11	115.7 (3)	C22—C20—C21	116.0 (2)
C5—C6—C7	128.8 (3)	O2—C20—H20	108.8
C11—C6—C7	115.5 (3)	C22—C20—H20	108.8
C8—C7—C6	120.6 (3)	C21—C20—H20	108.8
C8—C7—H7	119.7	O4—C21—C20	108.5 (2)
C6—C7—H7	119.7	O4—C21—C17	108.0 (2)
C7—C8—C9	122.9 (3)	C20—C21—C17	109.7 (2)
C7—C8—H8	118.5	O4—C21—H21	110.2
C9—C8—H8	118.5	C20—C21—H21	110.2
C10—C9—C8	118.7 (3)	C17—C21—H21	110.2
C10—C9—H9	120.6	O5—C22—C20	108.6 (2)
C8—C9—H9	120.6	O5—C22—H22A	110.0
C9—C10—C11	118.2 (2)	C20—C22—H22A	110.0
C9—C10—C12	133.3 (2)	O5—C22—H22B	110.0
C11—C10—C12	108.51 (19)	C20—C22—H22B	110.0
C2—C11—C10	113.4 (2)	H22A—C22—H22B	108.3
C2—C11—C6	122.6 (2)	C13—N1—C12	120.3 (2)
C10—C11—C6	124.0 (2)	C13—N1—C16	106.8 (2)
N1—C12—C10	114.91 (19)	C12—N1—C16	107.92 (19)
N1—C12—C1	103.87 (19)	C19—O2—C20	119.44 (19)
C10—C12—C1	102.06 (19)	C23—O5—C23'	14.1 (14)
N1—C12—C18	106.01 (17)	C23—O5—C22	118.9 (4)
C10—C12—C18	116.43 (19)	C23'—O5—C22	117.1 (7)
C1—C12—C18	113.00 (19)	C25'—O4—C25	14.2 (14)
N1—C13—C14	102.0 (2)	C25'—O4—C21	127.9 (12)
N1—C13—H13A	111.4	C25—O4—C21	113.8 (5)
C14—C13—H13A	111.4	O6—C23—O5	121.3 (7)
N1—C13—H13B	111.4	O6—C23—C24	120.6 (7)
C14—C13—H13B	111.4	O5—C23—C24	117.9 (7)
H13A—C13—H13B	109.2	O6'—C23'—O5	122.1 (12)
C15—C14—C13	102.6 (3)	O6'—C23'—C24'	131.5 (16)
C15—C14—H14A	111.2	O5—C23'—C24'	102.8 (13)
C13—C14—H14A	111.2	C23'—C24'—H24D	109.5
C15—C14—H14B	111.2	C23'—C24'—H24E	109.5
C13—C14—H14B	111.2	H24D—C24'—H24E	109.5
H14A—C14—H14B	109.2	C23'—C24'—H24F	109.5
C14—C15—C16	105.0 (2)	H24D—C24'—H24F	109.5
C14—C15—H15A	110.7	H24E—C24'—H24F	109.5
C16—C15—H15A	110.7	O7—C25—O4	125.4 (8)
C14—C15—H15B	110.7	O7—C25—C26	121.5 (8)
C16—C15—H15B	110.7	O4—C25—C26	113.2 (8)
H15A—C15—H15B	108.8	O7'—C25'—O4	114 (2)
N1—C16—C17	105.9 (2)	O7'—C25'—C26'	134.7 (18)
N1—C16—C15	104.8 (2)	O4—C25'—C26'	109.8 (16)
C17—C16—C15	117.1 (2)	C25'—C26'—H26D	109.5
N1—C16—H16	109.6	C25'—C26'—H26E	109.5
C17—C16—H16	109.6	H26D—C26'—H26E	109.5
C15—C16—H16	109.6	C25'—C26'—H26F	109.5
C21—C17—C16	116.3 (2)	H26D—C26'—H26F	109.5
C21—C17—C18	110.6 (2)	H26E—C26'—H26F	109.5
			
O1—C1—C2—C3	−3.5 (5)	N1—C12—C18—C17	−6.9 (2)
C12—C1—C2—C3	176.4 (3)	C10—C12—C18—C17	−136.1 (2)
O1—C1—C2—C11	173.5 (3)	C1—C12—C18—C17	106.2 (2)
C12—C1—C2—C11	−6.6 (3)	C17—C18—C19—O3	171.5 (2)
C11—C2—C3—C4	−0.7 (4)	C12—C18—C19—O3	−66.2 (3)
C1—C2—C3—C4	176.0 (3)	C17—C18—C19—O2	−5.7 (3)
C2—C3—C4—C5	−0.5 (5)	C12—C18—C19—O2	116.7 (2)
C3—C4—C5—C6	0.8 (5)	O2—C20—C21—O4	174.2 (2)
C4—C5—C6—C11	0.1 (4)	C22—C20—C21—O4	54.5 (3)
C4—C5—C6—C7	−179.0 (3)	O2—C20—C21—C17	−67.9 (3)
C5—C6—C7—C8	−179.5 (3)	C22—C20—C21—C17	172.3 (2)
C11—C6—C7—C8	1.5 (4)	C16—C17—C21—O4	48.0 (3)
C6—C7—C8—C9	−1.5 (5)	C18—C17—C21—O4	167.1 (2)
C7—C8—C9—C10	1.0 (4)	C16—C17—C21—C20	−70.2 (3)
C8—C9—C10—C11	−0.5 (4)	C18—C17—C21—C20	49.0 (3)
C8—C9—C10—C12	179.6 (3)	O2—C20—C22—O5	−69.4 (3)
C3—C2—C11—C10	−179.3 (2)	C21—C20—C22—O5	50.2 (3)
C1—C2—C11—C10	3.3 (3)	C14—C13—N1—C12	165.8 (2)
C3—C2—C11—C6	1.6 (4)	C14—C13—N1—C16	42.5 (3)
C1—C2—C11—C6	−175.8 (2)	C10—C12—N1—C13	32.2 (3)
C9—C10—C11—C2	−178.4 (2)	C1—C12—N1—C13	142.8 (3)
C12—C10—C11—C2	1.5 (3)	C18—C12—N1—C13	−97.9 (3)
C9—C10—C11—C6	0.7 (4)	C10—C12—N1—C16	154.9 (2)
C12—C10—C11—C6	−179.4 (2)	C1—C12—N1—C16	−94.5 (2)
C5—C6—C11—C2	−1.3 (4)	C18—C12—N1—C16	24.9 (2)
C7—C6—C11—C2	177.9 (2)	C17—C16—N1—C13	97.3 (2)
C5—C6—C11—C10	179.7 (3)	C15—C16—N1—C13	−27.0 (3)
C7—C6—C11—C10	−1.1 (4)	C17—C16—N1—C12	−33.3 (2)
C9—C10—C12—N1	−73.7 (4)	C15—C16—N1—C12	−157.7 (2)
C11—C10—C12—N1	106.4 (2)	O3—C19—O2—C20	168.5 (2)
C9—C10—C12—C1	174.6 (3)	C18—C19—O2—C20	−14.3 (3)
C11—C10—C12—C1	−5.3 (3)	C22—C20—O2—C19	175.71 (19)
C9—C10—C12—C18	51.1 (4)	C21—C20—O2—C19	50.5 (3)
C11—C10—C12—C18	−128.8 (2)	C20—C22—O5—C23	−154.5 (5)
O1—C1—C12—N1	67.3 (3)	C20—C22—O5—C23'	−138.6 (12)
C2—C1—C12—N1	−112.6 (2)	C20—C21—O4—C25'	−126 (3)
O1—C1—C12—C10	−173.0 (2)	C17—C21—O4—C25'	115 (3)
C2—C1—C12—C10	7.2 (3)	C20—C21—O4—C25	−123.5 (11)
O1—C1—C12—C18	−47.1 (3)	C17—C21—O4—C25	117.6 (11)
C2—C1—C12—C18	133.0 (2)	C23'—O5—C23—O6	−99 (4)
N1—C13—C14—C15	−41.0 (3)	C22—O5—C23—O6	−13.0 (10)
C13—C14—C15—C16	24.4 (3)	C23'—O5—C23—C24	85 (5)
C14—C15—C16—N1	0.1 (3)	C22—O5—C23—C24	171.8 (9)
C14—C15—C16—C17	−116.8 (3)	C23—O5—C23'—O6'	119 (6)
N1—C16—C17—C21	149.9 (2)	C22—O5—C23'—O6'	18 (3)
C15—C16—C17—C21	−93.8 (3)	C23—O5—C23'—C24'	−80 (4)
N1—C16—C17—C18	27.6 (2)	C22—O5—C23'—C24'	179.3 (14)
C15—C16—C17—C18	143.9 (2)	C25'—O4—C25—O7	171 (15)
C21—C17—C18—C19	−12.9 (3)	C21—O4—C25—O7	−1 (3)
C16—C17—C18—C19	113.0 (2)	C25'—O4—C25—C26	−9 (12)
C21—C17—C18—C12	−138.4 (2)	C21—O4—C25—C26	178.9 (10)
C16—C17—C18—C12	−12.4 (2)	C25—O4—C25'—O7'	12 (9)
N1—C12—C18—C19	−136.3 (2)	C21—O4—C25'—O7'	20 (6)
C10—C12—C18—C19	94.5 (2)	C25—O4—C25'—C26'	−180 (16)
C1—C12—C18—C19	−23.2 (3)	C21—O4—C25'—C26'	−171.1 (14)
